# From CD4-Based Initiation to Treating All HIV-Infected Adults Immediately: An Evidence-Based Meta-analysis

**DOI:** 10.3389/fimmu.2018.00212

**Published:** 2018-02-13

**Authors:** Aixin Song, Xinchao Liu, Xiaojie Huang, Kathrine Meyers, Djin-Ye Oh, Jianhua Hou, Wei Xia, Bin Su, Ni Wang, Xiaofan Lu, Huan Xia, Xiaodong Yang, Hui Chen, Hao Wu

**Affiliations:** ^1^Center for Infectious Diseases, Beijing You’an Hospital, Capital Medical University, Beijing, China; ^2^Infectious Diseases Department, Peking Union Medical College Hospital, Beijing, China; ^3^The Aaron Diamond AIDS Research Center, New York, NY, United States; ^4^School of Biomedical Engineering, Capital Medical University, Beijing, China

**Keywords:** HIV-infected adults, CD4+ T cell, early therapy, mortality, meta

## Abstract

**Background:**

The World Health Organization (WHO) Consolidated antiretroviral therapy (ART) guidelines set the CD4^+^ T-cell counts threshold to 500 cells/mm^3^ in 2013, and 2015 guidelines recommend treating all HIV-infected adults regardless of their CD4^+^ T-cell counts. To inform the decision-making around ART guidelines for people living with HIV, we systematically reviewed the literature to estimate differences in clinical benefits between individuals starting treatment with baseline CD4^+^ T-cell counts ≥500 cells/mm^3^ (early initiation) as compared to <500 cells/mm^3^ (deferred initiation).

**Methods:**

We systematically searched the electronic databases and abstracts for randomized controlled trials (RCT) and observational studies. Outcomes were mortality, AIDS progression, AIDS or death, immunologic recovery, and virologic suppression. We pooled data across studies and performed analyses of effect sizes.

**Results:**

We identified 13 studies comparing early and deferred treatment. The pooled risk ratio (RR) of mortality of 11 observational studies was 0.90 (95% CI 0.82–0.99), with moderate heterogeneity (*I*^2^ = 53%). The pooled RR for progression to AIDS from two observational studies was 0.77 (95% CI 0.47–1.24). Five observational studies found a pooled RR of death or AIDS of 0.94 (95% CI 0.93–0.95). For the outcome of immunologic recovery, defined as CD4^+^ T-cell counts reaching at least 800 cells/mm^3^ after ART, one observational study found early initiation of ART had an HR (hazard ratio) of 2.39 (95% CI 1.93–2.96). The pooled RR of viral suppression (a viral load <50 copies/ml) after 9 months from one cohort was 1.04 (95% CI 0.99–1.09).

**Conclusion:**

Mortality risk and risk for AIDS appear to be reduced among people living with HIV with early initiation of ART, based on current WHO guidelines, as compared to those with deferred initiation of ART (<500 cells/mm^3^).

## Introduction

Optimal timing of antiretroviral therapy (ART) initiation for people living with HIV and AIDS (PLWHA) is essentially a risk-benefit question (1). In the mid-1990s, with the advent of combined ART, the prognosis of the disease improved tremendously (2, 3). Since then, different thresholds for initiating ART have been proposed over time (CD4 counts of 200, 350, and 500 cells/mm^3^). There is a consensus that CD4 cell counts measurements have been at the core of understanding disease progression and decisions regarding initiating treatment in AIDS patients. The measurements are used to prevent opportunistic infections as well as to monitor the treatment response (4).

During the first 10 years of ART use, the ART-initiation threshold fluctuated. After a short period in the late 1990 s, Department of Health and Human Services recommended to start treatment for ≤500 cells/mm^3^; however, this recommendation came in the absence of randomized trial evidence and was not taken up in clinical practice (5). The threshold for treating asymptomatic adults dropped below 200 cells/mm^3^ at the beginning of the 2000s (5). This shift was mainly due to taking into account the cumulative risk of ART drug-related toxicity. During that period, randomized clinical trials of structured treatment interruption after immune recovery above 350 cells/mm^3^ were carried out as a way to minimize ARV-related toxicity (6, 7). However, these studies concluded that the damage of HIV is more severe than that of antiretroviral drugs.

Between 2006 and 2009, World Health Organization (WHO) raised the CD4 threshold to 350 cells/mm^3^ based on research demonstrating that ART initiation at this threshold reduced mortality, disease progression and serious adverse events (8). The 2013 WHO consolidated ART guidelines further set the threshold to 500 cells/mm^3^ or less. Finally, between 2012 and 2015, all international guidelines took the last step and recommended treating all HIV-infected adults, regardless of CD4 counts (9–11). Of note, in resource-limited countries, health care infrastructure and the availability of antiretroviral drugs may be more limited, and in that case ART remains prioritized for patients with the most advanced disease.

Meanwhile, The Joint United Nations Programme on HIV/AIDS (UNAIDS) set international goals for 2020 termed the “90–90–90 targets” to curb the HIV epidemic: 90% of PLWHA know their HIV status, 90% of people who know their HIV status access treatment and 90% of people on treatment have suppressed viral loads (12). The second “90” (treatment), can realistically only be accomplished with early ART initiation. Furthermore, ART does not just reduce mortality in the treated individuals, it also reduces the number of people with a detectable viral load. That finding, as expected, show that early ART initiation helps to accomplish the third “90” (suppression) of 90–90–90 targets.

The benefit of treating all HIV-infected adults, rather than deferring treatment until CD4^+^ T cell counts drops below 500 cells/mm^3^ or even lower, was initially demonstrated in a large cohort study (13), followed by two randomized controlled trials (RCTs) (14, 15). The goal of this meta-analysis was to comprehensively review the available observational study data for evidence supporting the benefits of early ART. We systematically reviewed the literature to estimate differences in risk of disease progression between people living with HIV-1 whose CD4^+^ T cell counts at ART initiation was 350–499 cells/mm^3^ and patients whose CD4^+^ T cell counts at ART initiation was ≥500 cells/mm^3^. The primary outcome was mortality, a composite outcome that included AIDS progression, AIDS or death, immunological recovery and virological suppression. We conducted a meta-analysis to evaluate the outcomes of patients initiating ART at different CD4 cell counts.

## Methods

The review was registered in the International Prospective Register of Systematic Reviews (PROSPERO, http://www.crd.york.ac.uk/PROSPERO): CRD42017072465.

### Data Sources and Search Strategy

We followed the meta-analysis of observational studies in epidemiology guidelines for study procedures (16). We searched PubMed, MEDLINE, Web of Science and Embase for journal articles published between 1 January 2000 and 31 May 2017 which reported CD4 counts at ART initiation among HIV-infected adults. The search strategy included Medical Subject Heading terms and a range of relevant keywords, including “HIV,” “ART,” “early/deferred therapy,” and “CD4.” We included studies and investigations in HIV-1-infected patients who began ART with CD4^+^ T cell counts less than 500 cells/mm^3^ and at least 500 cells/mm^3^. Randomized trials, prospective and retrospective cohorts, and routine clinic cohorts were eligible for inclusion. There were no geographic or age restrictions. The search was limited to peer-reviewed and English-written journal articles.

### Study Selection and Data Extraction

We first screened titles and abstracts to identify relevant articles for inclusion. To exclude duplicate references, we imported search results into bibliographic citation management software (EndNote X7). Two authors reviewed the titles, abstracts, and descriptor entries of the remaining citations to identify potentially eligible reports independently. We obtained full-text articles for references that were considered potentially meeting the inclusion criteria. We reviewed these full-text articles and applied the inclusion criteria to determine the eligibility or ineligibility of each study. We planned to resolve any differences of opinion through discussion and, if necessary, we would have a neutral third party arbiter. After determining articles for inclusion, we performed an examination and extracted data from each study.

Information extracted from articles included article author, publication year, type of screening test, sample size, CD4^+^ T-cell counts, and primary outcomes (mortality and AIDS progression).

### Statistical Analysis and Data Synthesis

We combined data across studies and estimated summary treatment effect sizes. We performed meta-analyses using Review Manager 5.3. We used published estimated relative risks (RRs) if these data were provided in study reports. If not available, we calculated RRs for outcomes and the 95% confidence interval (CI). Subgroup analysis was also performed to evaluate the moderate effect of study location on mortality due to sufficient comparisons. Due to the clinical heterogeneity between study designs and populations, we chose fixed-effects or random-effects model based meta-analysis according to *I*^2^ for combining data. We used *I*^2^ to quantify the degree of heterogeneity (17, 18). Estimates of *I*^2^ were defined as the percentage of variability in effect estimates due to heterogeneity rather than chance.

### Assessment of Evidence Quality

We used the Newcastle Ottawa Scale to assess quality and risk of bias in the nonrandomized studies (Table 1) (19). This scale judges three general areas: selection of study groups, comparability of groups, and ascertainment of outcomes (in the case of cohort studies). We assessed the quality of evidence from the relevant studies for each outcome. Because of the particularity of the study, the literature we collected are observational studies. Evidence from these studies starts at low, but can be upgraded if the sample size and magnitude of treatment effect are very large, if there is an obvious dose–response relationship or if all possible mixed factors would decrease the magnitude of the treatment effect (20). Evidence from observational studies can also be downgraded. To minimize the problem of overlap, we removed one smaller sample study from the final analysis, thereby avoiding the possibility of double-counting patient populations.

**Table 1:** Summary of critical appraisal of included studies using the Newcastle–Ottawa Quality Assessment Scale for observational studies.

Reference	Selection				Comparability	Outcome		

	Representativeness	Selection of non-exposed	Ascertainment of exposure	Demonstration outcome not present at start	Comparability of cohorts	Assessment of outcome	Follow up long enough	Adequate follow-up rate (≥80%)
Lodi et al. (21)	☆	☆	☆	☆	☆☆	☆	☆	☆
Lima et al. (22)	☆	☆	☆	☆	☆☆	☆	☆	☆
Sterne et al. (23)	☆	☆	☆	☆	☆☆	☆	☆	☆
May et al. (24)	☆	☆	☆	☆	☆☆	☆	☆	☆
Nsanzimana et al. (25)	☆	☆	☆	☆	☆☆	☆	☆	−
Gabillard et al. (26)	☆	☆	☆	☆	☆☆	☆	☆	☆
Kitahata et al. (13)	☆	☆	☆	☆	☆☆	☆	☆	☆
Garcia et al. (27)	☆	☆	☆	☆	☆☆	☆	☆	☆
Gras et al. (28)	☆	☆	☆	☆	☆☆	☆	☆	−
Ray et al. (29)	☆	☆	☆	☆	☆☆	☆	☆	☆
Cain et al. (30)	☆	☆	☆	☆	☆☆	☆	☆	☆
Palella et al. (31)	☆	☆	☆	☆	☆☆	☆	☆	☆

## Results

**Figure 1:**
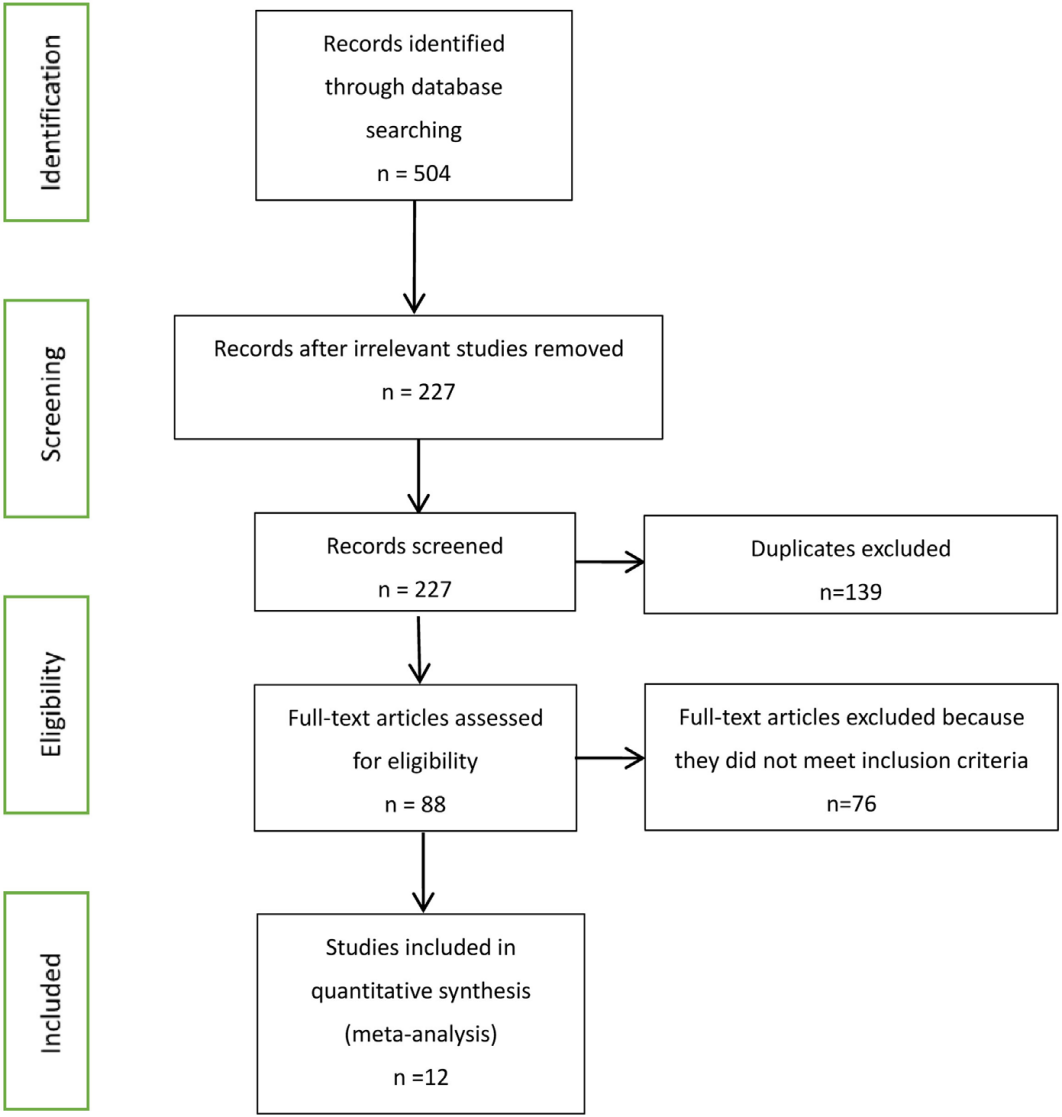
Flow chart of selection process for the inclusion of studies.

Our searches identified 504 published journal articles. After review of the title and abstract, we selected 12 full-text articles that met our inclusion criteria (Figure 1). Two RCT studies failed to meet all inclusion criteria, because they focused on HIV-infected patients who began ART with CD4^+^ T cell counts less than 350 cells/mm^3^ and more than 500 cells/mm^3^. The remainder were observational studies (13, 21–31) (Table 2) with a variety of outcomes, including mortality, progression to AIDS, progression to AIDS or death, serious non-AIDS events, CD4^+^ T-cell counts increase, virologic suppression, and virologic failure.

**Table 2:** Characteristics of included studies.

Reference	Methods	Period	Sample size	Location	Participants	Intervention
Lodi et al. (21)	Prospective cohort studies	2000–2013	55,826	Europe and the United States	HIV-infected patients	Initiate ART with CD4 counts >500 cells/ml

Lima et al. (22)	Retrospective cohort study	2000–2012	4,120	British Columbia and Canada	HIV-infected patients	Initiate ART with CD4 counts >500 cells/ml

Sterne et al. (23)	Collaborative analysis of 18 cohort studies	1998–2008	24,444	Europe and North America	HIV-infected patients	Initiate ART with CD4 counts 451–550 cells/ml

May et al. (24)	Collaborative analysis of 18 cohort studies	1996–2001	37,495	Europe and North America	HIV-infected patients	Initiate ART with CD4 counts ≥500 cells/ml

Nsanzimana et al. (25)	Nationwide cohort study	1997–2014	50,147	Rwanda	HIV-infected patients	Initiate ART with CD4 counts ≥500 cells/ml

Gabillard et al. (26)	Pooled data from 13 research cohorts	1998–2008	3,917	Five sub-Saharan African (Benin, Burkina Faso, Cameroon, Cote d’Ivoire, and Senegal) and two Asian (Cambodia and Laos)	HIV-infected adults (≥18 years)	Initiate ART with CD4 counts 501–650 cells/ml

Kitahata et al. (13)	Pooled data from 22 cohort studies	1996–2005	8,362	Canada and United States of America	HIV-infected patients	Initiate ART with CD4 counts >500 cells/ml

Garcia et al. (27)	Cohort study	1996–2003	861	Spain	HIV-infected patients	Initiate ART with CD4 counts >500 cells/ml

Gras et al. (28)	Multicentre cohort study	1996–2004	5,299	The Netherlands	HIV-infected patients	Initiate ART with CD4 counts >500 cells/ml

Ray et al. (29)	Prospective observational data of 12 cohort studies	1997–2006	62,760	France, The Netherland, Spain, Switzerland, United Kingdom, United States of America	HIV-infected patients	Initiate ART with CD4 counts ≥500 cells/ml

Cain et al. (30)	Prospective observational data of 12 cohort studies	1996–2009	8,392	France, The Netherland, Spain, Switzerland, United Kingdom, United States of America	HIV-infected patients	Initiate ART with CD4 counts >500 cells/ml

Palella et al. (31)	Cohort study	1994–2001	1,464	United States of America	HIV-infected patients	Initiate ART with CD4 counts 501–750 cells/ml

### Mortality (350–499 vs. ≥500 cells/mm^3^)

**Figure 2:**
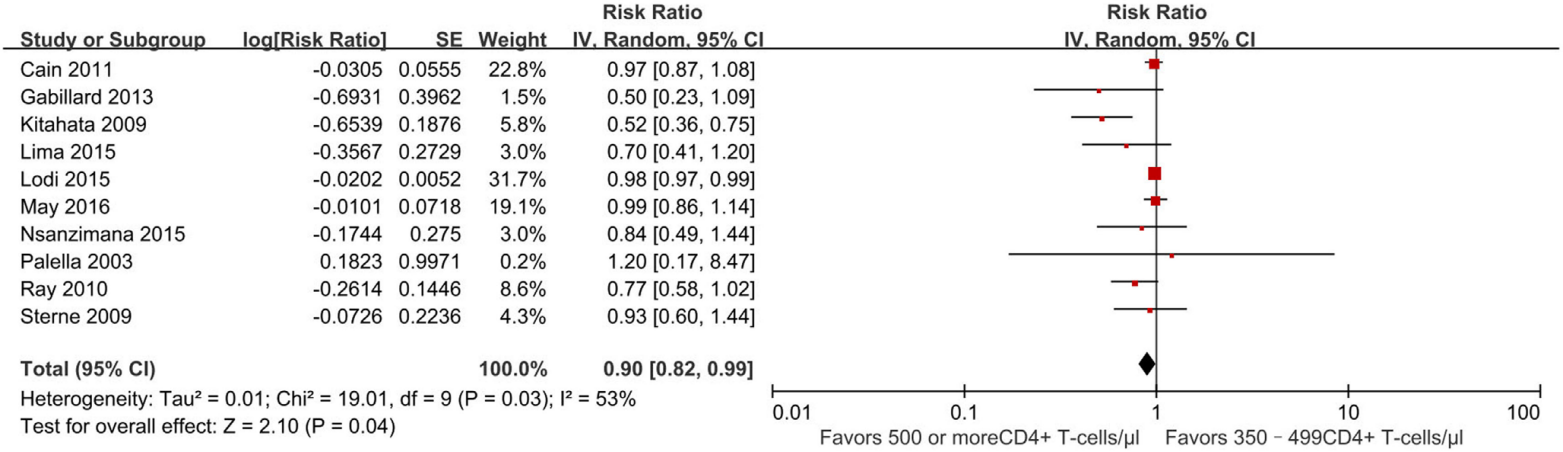
Forest plots of mortality. CI, confidence interval; DF, degrees of freedom; IV, inverse variance.

There were 10 studies (13, 21–26, 29–31) reporting a mortality outcome (Figure 2). One study reported a slightly increased risk of death in patients who initiated ART at CD4^+^ T-cell counts of at least 500 cells/mm3 (31), whereas nine studies reported a decreased risk of death (pooled RR 0.90, 95% CI 0.82–0.99), which was statistically significant for two of these nine studies (13, 21).

**Figure 3:**
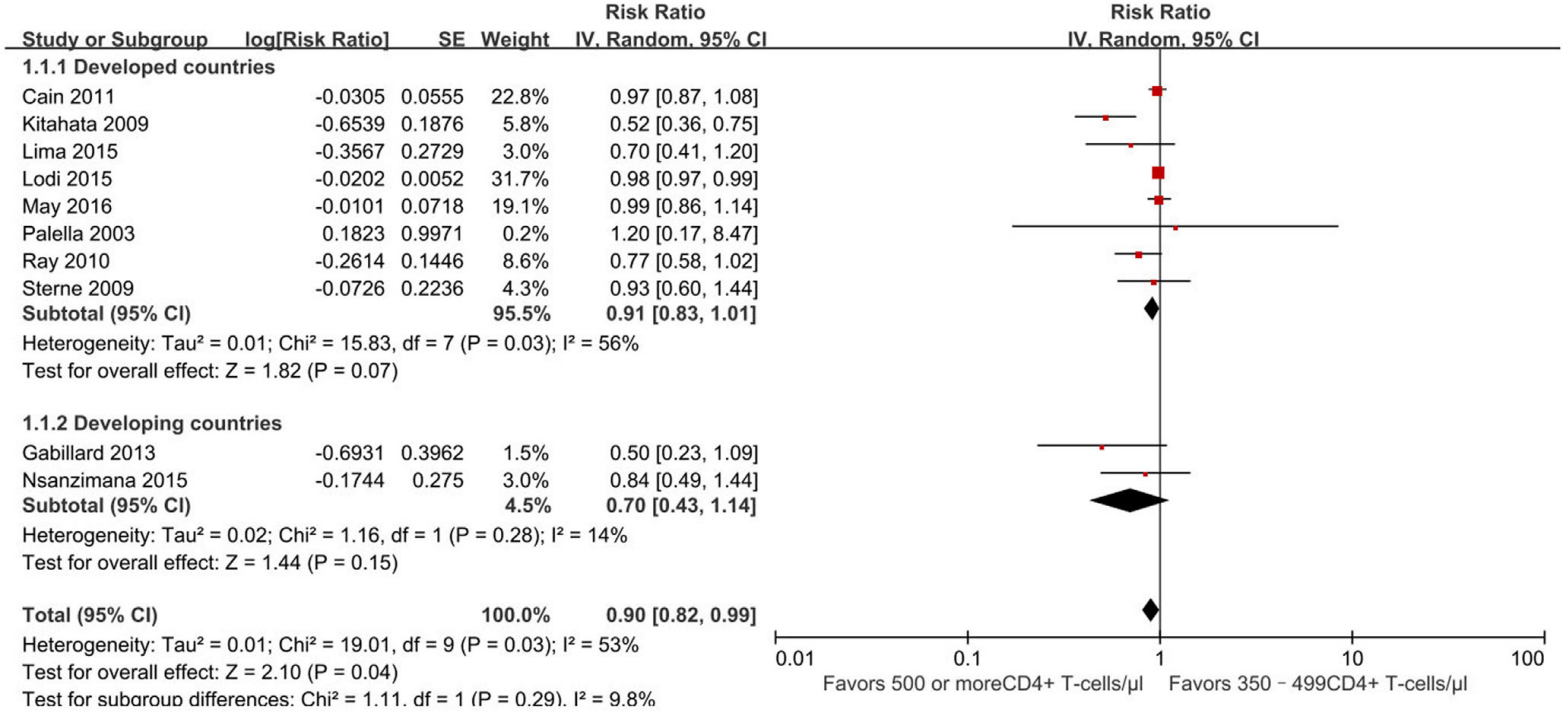
Forest plots of subgroup analysis of mortality. CI, confidence interval; DF, degrees of freedom; IV, inverse variance.

Furthermore, we conducted a subgroup analysis of developed and developing countries separately based on the location of these 10 studies (Figure 3). Further analysis focus on whether developing countries with a higher burden of infectious diseases have different outcomes than developed countries. Of 10 studies, 8 were conducted in developed countries (13, 21–24, 29–31) and 2 in developing countries (25, 26). Subgroup analysis revealed no statistically significant difference between the two groups (RR for developing country, 0.91, 95% CI 0.83–1.01, developed country, 0.70, 95% CI 0.43–1.14, *p* = 0.29).

The evidence quality of the observational literature which reported mortality was poor due to clinical heterogeneity and inconsistency of findings across the 10 studies. However, the evidence quality of the non-overlapping studies was moderate because of a large effect size.

### AIDS Progression (350–499 vs. ≥500 cells/mm^3^)

**Figure 4:**
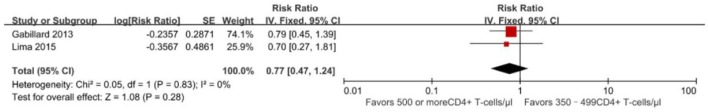
Forest plots of AIDS progression. CI, confidence interval; DF, degrees of freedom; IV, inverse variance.

Compared to ART initiation at 350–499 CD4^+^ T cells/mm^3^, initiation of ART at ≥500 cells/mm^3^ was found to be associated with decreased risk of developing AIDS-defining opportunistic infections in a pooled analysis of two non-overlapping studies: RR = 0.77 (95% CI 0.47–1.24) (22, 26) (Figure 4), there are no statistically significant effects (*p* = 0.28). The evidence quality of the observational literature was very poor due to small number of events. None of these two studies reported adjusted estimates, so that the risk of bias from unadjusted estimates has been increased.

### Mortality and/or AIDS-free Survival (350–499 vs. ≥500 cells/mm^3^)

**Figure 5:**
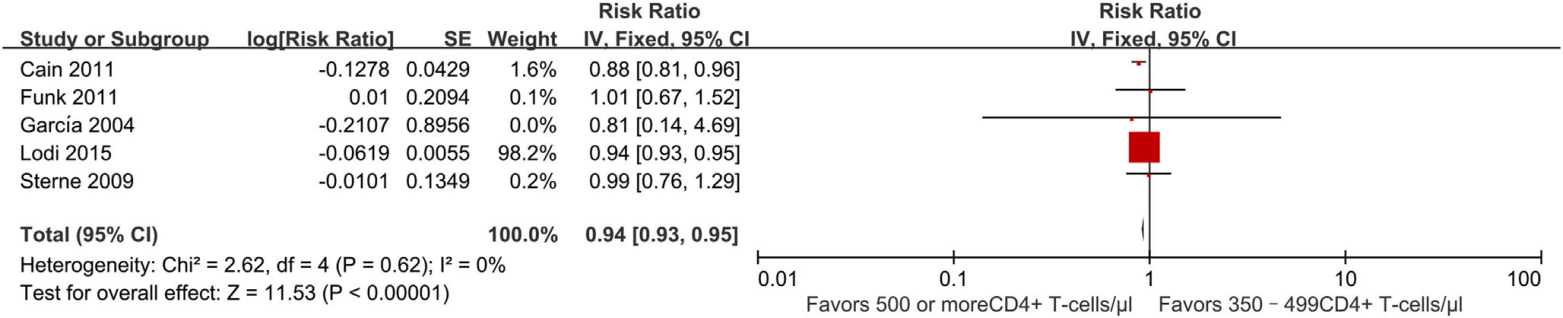
Forest plots of AIDS progression or death. CI, confidence interval; DF, degrees of freedom; IV, inverse variance.

Four studies (21, 23, 27, 30) evaluated the effect of early vs. deferred ART initiation. All of these found a decreased risk of progression to AIDS and/or death among persons who initiated ART early as compared to those who deferred treatment. The RR was 0.94 (95% CI 0.93–0.95) (Figure 5). Two studies found a statistically significant effect (21, 30). Both were based on data from the HIV-CAUSAL collaboration (21) of cohorts in Europe and the United States, for which sample size is large; they found a slightly lower risk of AIDS progression among patients beginning treatment at CD4^+^ T-cell counts of ≥500 cells/mm^3^ compared to those with deferred treatment.

These observational studies were consistent in the identification of treatment effect. However, the quality of the literature for this outcome was poor due to imprecision owing to its observational status and the lack of strength.

### Immunologic Recovery

**Figure 6:**
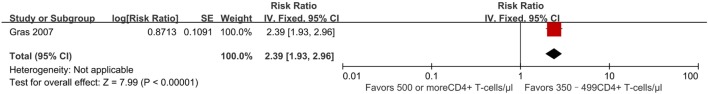
Forest plots of immunologic recovery. CI, confidence interval; DF, degrees of freedom; IV, inverse variance.

Outcome used to determine immune recovery in this study included CD4^+^ T-cell counts reaching at least 800 cells/mm^3^ after initiation of ART. One study from the AIDS Therapy Evaluation Project and Netherlands (ATHENA) national observational HIV cohort (28) estimated the effect of early vs. deferred treatment on CD4^+^ T-cell counts reaching 800 cells/mm^3^ or more after 7 years of ART. It found that compared to patients who began ART at 350–499 CD4^+^ T cell/mm^3^, those who began ART at ≥500 CD4^+^ T cells/mm^3^ had an HR of 2.39 (95% CI 1.93–2.96) (Figure 6). This outcome was statistically significant (*p* < 0.00001), although evidence quality of this literature was rated as poor owing to its observational nature and overlap of patient populations between studies.

### Virologic Suppression

**Figure 7:**
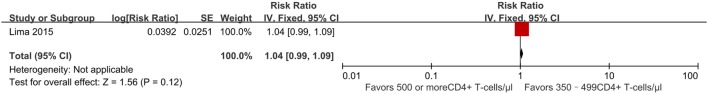
Forest plot of virologic suppression. CI, confidence interval; DF, degrees of freedom; IV, inverse variance.

Successful treatment requires a prolonged and profound virological response, so a key consideration in deciding when to initiate ART is whether it can achieve viral suppression. We modeled the probability of maintaining viral suppression (a viral load <50 copies/ml) over time. In a retrospective cohort study performed between 2000 and 2012, initiating ART at CD4^+^ T-cell counts of ≥500 cells/mm^3^ was associated with a better effect of achieving viral suppression after 9 months (RR 1.04, 95% CI 0.99–1.09) (Figure 7). The percentage of individuals displaying viral suppression was 89% in those initiating ART ≥500 cells/mm^3^, compared to 86% in those initiating ART at 350–499 cells/mm^3^ (22). The quality of this literature for outcomes was high with no observed study limitations.

## Discussion

World Health Organization recommendations to initiate ART for persons switching from CD4 counts ≤500 cells/mm^3^ (32) to treating all HIV-infected adults regardless of their CD4 counts (9–11) have spurred debate about the benefits of adopting this guide as well as discussion about the extent to which existing health systems can accommodate the expansion of treatment availability (33–35).

There is a strong association between CD4^+^ T-cell counts at ART initiation and all outcomes investigated in this study. We found evidence to suggest immediate ART initiation (with baseline CD4 ≥500 cells/mm^3^) can reduce the risk of mortality and progression to AIDS, can improve the likelihood of immunologic recovery (CD4^+^ T-cell counts reaching 800 cells/mm^3^ or more after ART) and can increase viral suppression at 9 months. There was generally consistent agreement between the large observational literature although few individual studies did show opposite results (31).

Our findings indicate that, compared with delayed treatment strategies, immediate universal ART slightly prolongs survival, decreases risk of progression to AIDS/death, and we tentatively put forward it also increases the percentage of individuals who achieve virological suppression. They render further support to the evidence from three major published trials, HPTN 052, Temprano ANRS 12136 and START, which demonstrated that there is a health benefit from immediate initiation of ART, and that this is all the more true for those living in low resource settings. In fact, HIV-infected persons are now recommended to initiate ART regardless of CD4 counts all over the world, and the objective of ART clearly turns to suppressing viral replication and preventing inflammation and immune deficiency (1). Observational studies will continue to play a role in understanding the long-term effects of initiating ART at high CD4 counts in conventional clinical settings. Moreover, our study indicates a similar decreased mortality in developing and developed countries. However, the reality is that treatment roll-out to all those in need of ART remains far from complete, especially in countries with a high HIV burden (36). Broadening treatment eligibility directly leads to an increase in the number of individuals treated, while the vast majority of the 35 million PLWHA are in resource-limited settings (37). Improving the timeliness of ART initiation will also require significant additional financial investment (38). This is a challenge for developing countries. One question is whether in HIV elite controllers, the benefits of early treatment actually do outweigh the risks (39, 40). In this respect, one needs to explore the relevant question of “when not to start ART immediately” (40).

The achievement of the goals of “90-90-90 targets” requires immediate treatment linked to viral suppression. According to this recommendation of starting ART immediately in HIV-infected adults, countries should adopt and implement this policy to accelerate progress toward the goals and eventually reach epidemic control (41). However, to achieve early ART and eliminate AIDS globally will require constant effort and substantially higher levels of treatment coverage, a near doubling of the number of people on treatment today (42).

Several limitations of this meta-analysis should be addressed. First, as with all systematic reviews, we are limited by the sensitivity of our search and our ability to identify relevant studies. Second, our estimates come from high-income countries in Europe and the USA, with only a small contribution from Africa, Asia, and South America, which may somewhat limit the outcome effects. Third, a meta-analysis of observational studies is inherently limited because observational cohort studies may provide a relative lower quality of evidence than randomized controlled clinical trials. Furthermore, it is possible that the data density such as frequency of follow-up visits and clinical assessment between these periods may have affected our results. There might be additional potential limitations given the English language bias.

In conclusion, our findings lead us to argue that more resources should be dedicated to both facilitating an earlier diagnosis of HIV and expediting ART initiation as well as retaining in care of those newly diagnosed (43). This does not only mean more tests and more drugs, but it also requires more caretakers to support those living with HIV and AIDS and to help them remain in care once they have started treatment (38).

## Author Contributions

AS, XCL, XH, and HW conceived and designed the protocol and study. JH, WX, NW, HX, and XY identified studies to be screened. XH, KM, D-YO, BS, and XFL identified studies for eligibility, extracted data and assessed the methodologic quality of included studies. AS performed the analysis with assistance from HC and HW. All authors read and approved the final manuscript.

## Conflict of Interest Statement

The authors declare that the research was conducted in the absence of any commercial or financial relationships that could be construed as a potential conflict of interest.
